# A combination of veno-arteriovenous ECMO and Impella (VAVEcpella) as a rescue strategy for severe streptococcal toxic shock syndrome with cardiopulmonary failure: A case report

**DOI:** 10.1097/MD.0000000000043741

**Published:** 2025-08-08

**Authors:** Wataru Otsuru, Masaaki Nishihara, Kiwamu Hatakeyama, Takeshi Iyonaga, Takeo Fujino, Takafumi Sakamoto, Yuji Shono, Kohtaro Abe, Akira Shiose, Tomohiko Akahoshi

**Affiliations:** a Emergency and Critical Care Center, Kyushu University Hospital, Fukuoka, Japan; b Department of Cardiovascular Medicine, Faculty of Medical Sciences, Kyushu University, Fukuoka, Japan; c Department of Cardiovascular Surgery, Kyushu University Hospital, Fukuoka, Japan.

**Keywords:** Impella, sepsis-induced cardiomyopathy, septic shock, streptococcal toxic shock syndrome, veno-arteriovenous extracorporeal membrane oxygenation

## Abstract

**Rationale:**

Streptococcal toxic shock syndrome (STSS) is an invasive *Streptococcus* pyogenes infection characterized by hypotension and multiple organ failure with rapid progression and high mortality. Although extracorporeal membrane oxygenation (ECMO) has been used in adults with STSS, mortality remains high and optimal mechanical circulatory support is controversial. Veno-arterial ECMO has specific complications in severe cardiopulmonary failure, including differential hypoxia and increased left ventricular end-diastolic pressure due to retrograde flow.

**Patient concerns:**

A 51-year-old man presented to the emergency department with fever and dyspnea, progressing rapidly from an initially diagnosed upper respiratory tract infection to severe respiratory distress and refractory shock requiring oxygen supplementation and vasopressor support.

**Diagnoses:**

The patient was diagnosed with STSS, which manifested as septic shock with severe cardiopulmonary compromise.

**Interventions:**

We implemented a combined approach using veno-arteriovenous ECMO (V-AV ECMO) and Impella CP^®^ support (veno-arteriovenous extracorporeal membrane oxygenation and Impella [VAVEcpella]). This strategy provided oxygenated blood to the right heart while achieving left ventricular unloading. This was done in conjunction with appropriate antibiotic therapy and source control measures.

**Outcomes:**

The novel VAVEcpella approach successfully supported the patient through severe cardiopulmonary failure secondary to STSS-induced septic shock. To our knowledge, this is the first reported case of VAVEcpella implementation specifically for the management of STSS.

**Lessons:**

The VAVEcpella approach (combined V-AV ECMO and Impella support) may represent a viable rescue strategy for patients with severe cardiopulmonary failure secondary to septic shock, such as STSS, where traditional support methods have shown limited success.

## 1. Introduction

Streptococcal infections are common, ranging from mild to severe disease. Streptococcal toxic shock syndrome (STSS), primarily due to group A *Streptococcus*, known as *Streptococcus pyogenes*, with an incidence of 2 to 4/1,00,000,^[1]^ progresses rapidly and can cause soft tissue necrosis, acute renal failure, acute respiratory distress syndrome, disseminated intravascular coagulation, and a mortality rate of up to 48%.^[2]^ Early diagnosis and multidisciplinary management – including intensive care, infection control, surgery and antibiotics-are essential for patients with STSS.^[3]^

Sepsis-induced cardiomyopathy (SICM), a transient form of global biventricular dysfunction with LV dilatation and poor response to fluids and catecholamines, is reversible in 7 to 10 days with a prevalence of 10% to 70% in septic patients.^[4,5]^ In STSS, a cohort study in Barcelona reported that 76.9% of patients experienced SICM, with 53.8% requiring veno-arterial extracorporeal membrane oxygenation (V-A ECMO).^[6]^

**Figure 1.**
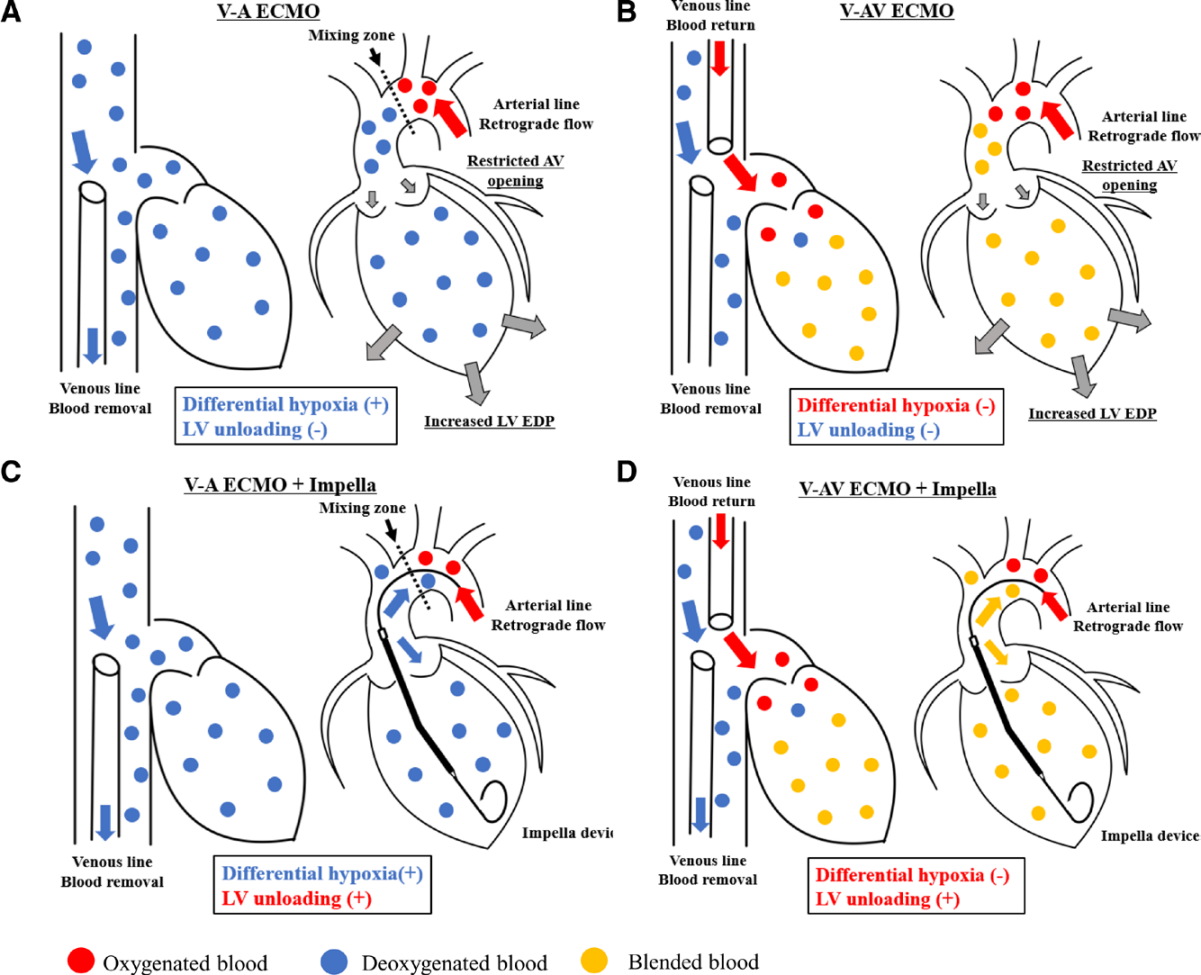
Schematic of various combinations of mechanical circulatory support devices in the presence of both cardiac and respiratory dysfunction. Right heart and left heart are described separately. (A) V-A ECMO: The mixing zone is determined by cardiac output and return blood flow through the arterial line. In case of severe LV failure and respiratory failure, differential hypoxia is induced. In addition, the returned blood flow increases LVEDP and restricts aortic valve opening. (B) V-AV ECMO: Oxygenated blood is returned to the right atrium, reducing the risk of differential hypoxia. (C) V-A ECMO+Impella: Impella helps circulate blood from the left ventricle to the ascending aorta, leading LV unloading. In case of biventricular failure, Impella is used as an LV vent. (D) V-AV ECMO+Impella: Antegrade oxygenated blood circulates from the LV to the ascending aorta, reducing the risk of differential hypoxia and leading LV unloading. EDP = end diastolic pressure, LV = left ventricular, V-A ECMO = veno-arterial extracorporeal membrane oxygenation, V-AV ECMO = veno-arteriovenous extracorporeal membrane oxygenation. Modified and adapted from Shimizu et al,^[10]^ Copy right (2020), with permission from Elsevier.

Mechanical circulatory support (MCS) for refractory septic shock in adults remains controversial.^[7]^ The meta-analysis discussing the use of V-A ECMO in septic patients suggests its effectiveness in those with SICM.^[8]^ Careful evaluation of cardiac function and respiratory status is essential for determining the most suitable ECMO configuration.^[9]^ In cases of severe cardiopulmonary dysfunction, V-A ECMO carries the risk of differential hypoxia and increased LV end-diastolic pressure. Differential hypoxia means the circulation of low oxygenated blood to the coronary and cerebral arteries via the native pulmonary pathway. Increased left ventricular end-diastolic pressure is caused by retrograde blood return from arterial line of ECMO, aggravates pulmonary congestion, and restricts the opening of aortic valve which leads left ventricular (LV) thrombus formation (Fig. 1A). To overcome these risks, the use of veno-arteriovenous ECMO (V-AV ECMO; Fig. 1B) or the Impella device (Fig. 1C) is being considered. The combination of V-AV ECMO and Impella, called veno-arteriovenous extracorporeal membrane oxygenation and Impella (VAVEcpella), offers the dual benefit of reducing differential hypoxia and providing effective LV unloading (Fig. 1D). The use of VAVEcpella has been reported in cases of cardiopulmonary arrest caused by acute coronary syndrome, myocarditis, and fatal arrhythmia.^[11,12]^

Here in, we first report a case of STSS with severe respiratory failure caused by pneumonia and circulatory failure caused by SICM, treated with VAVEcpella. We discuss the advantages of VAVEcpella support in respiratory and circulatory function, and consider the potential of using this novel MCS for severe cardiopulmonary failure secondary to septic shock.

## 2. Case presentation

**Figure 2.**
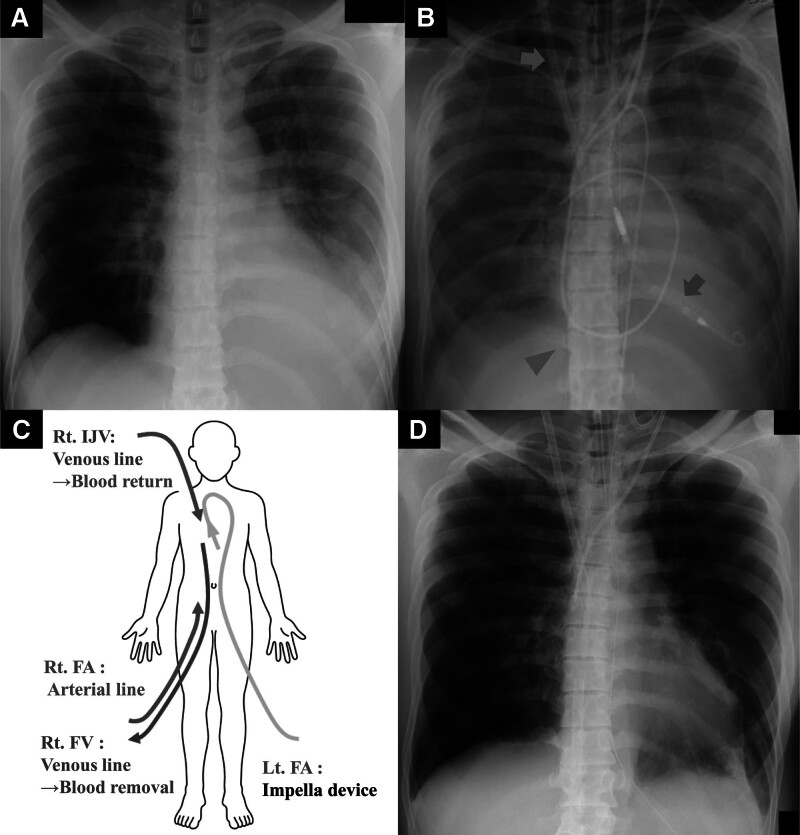
Sequential change in the chest radiographs and an illustration of VAVEcpella device system. (A) Chest x-ray on arrival showing pneumonia limited to the left lower lung field. (B) Rapid decrease in radiographic translucency over the entire lung field within 1 d, followed by VAVEcpella system placement. Red arrow indicates Impella CP^®^, blue arrow indicates venous line for blood return, blue arrowhead indicates venous line for blood removal. (C) An illustration of the VAVEcpella device system. (D) Improved lung field translucency after antibiotic therapy and prone positioning with mechanical circulatory support. FA = femoral artery, FV = femoral vein, IJV = internal jugular vein, VAVEcpella = veno-arteriovenous extracorporeal membrane oxygenation and Impella.

A 51-year-old man presented to the emergency department with fever and dyspnea. He reported having a fever 2 days and consulted a local physician. He was diagnosed with an upper respiratory tract infection and was prescribed antipyretics. On admission, he was febrile (37.0°C) with tachycardia (120 beats/min), hypotension (84/54 mm Hg), tachypnea (30 breaths/min), and hypoxemia (pO_2_ 70 Torr on 10 L of oxygen). Examination revealed bilateral lower lung rales and laboratory findings included elevated C-reactive protein (19.9 mg/dL) and lactate (38 mg/dL). Imaging revealed ground glass opacities and bilateral infiltrates (Fig. 2A). He was administered oxygen through a high-flow nasal cannula, lactated Ringer’s solution intravenously, and continuous intravenous infusion of norepinephrine for refractory vasodilatory shock.

**Figure 3.**
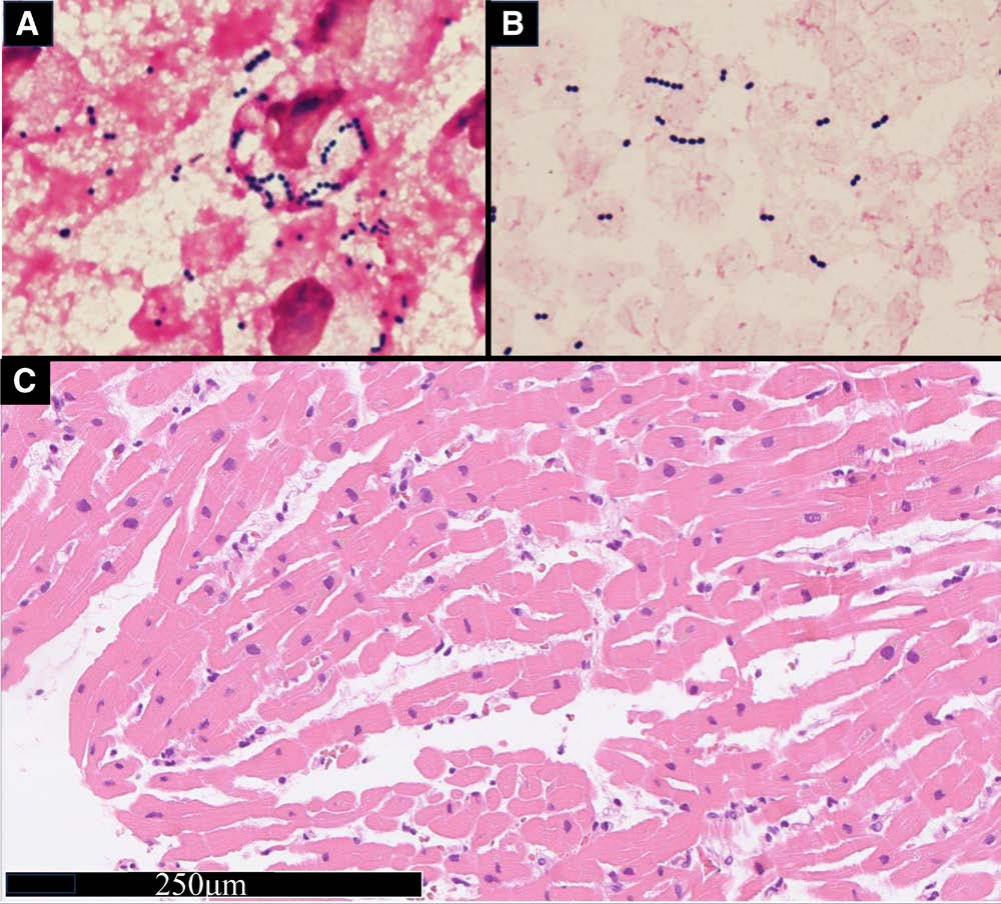
Images of sputum culture, blood culture (gram staining), and myocardial tissue (hematoxylin and eosin staining). Sputum and blood cultures were collected at the time of admission. Gram-positive streptococci were observed, along with evidence of phagocytosis by white blood cells in sputum (A), blood (B) culture (gram staining, magnification: ×1000). (C) Myocardial tissue showed mildly hypertrophic myocytes having irregular nuclei and lipofuscin granules. Minimal inflammatory infiltration was seen, but myocytic damage was not seen (hematoxylin and eosin staining, magnification: ×20).

He was diagnosed with septic shock secondary to severe pneumonia. He was admitted to the intensive care unit and administered meropenem because the causative bacteria were not identified. Due to prolonged circulatory failure secondary to septic shock, the continuous intravenous infusion of norepinephrine was increased to 0.2 μg/kg/min, vasopressin was administered at 0.02 unit/kg/h, and hydrocortisone 50 mg was given every 6 hours. Five hours after admission to the intensive care unit, the patient required intubation due to a significant deterioration in oxygenation (PaO_2_/FiO_2_ ratio: 70). Blood and sputum cultures were positive for *S pyogenes* 15 hours after presentation (Fig. 3A, B). A diagnosis of STSS was made, since this case fulfilled the diagnostic criteria established by the CDC (Centers for Disease Control and Prevention) in 2010 (hypotension, renal impairment [creatinine: 2.59 mg/dL], coagulopathy [platelets: 7.2 × 10⁶/L], liver involvement [alanine aminotransferase: 142 IU/L], and isolation of group A *Streptococcus*).^[13]^ Antibiotic regimen was changed from meropenem to ampicillin and clindamycin, and intravenous immunoglobulin was administered.

**Figure 4.**
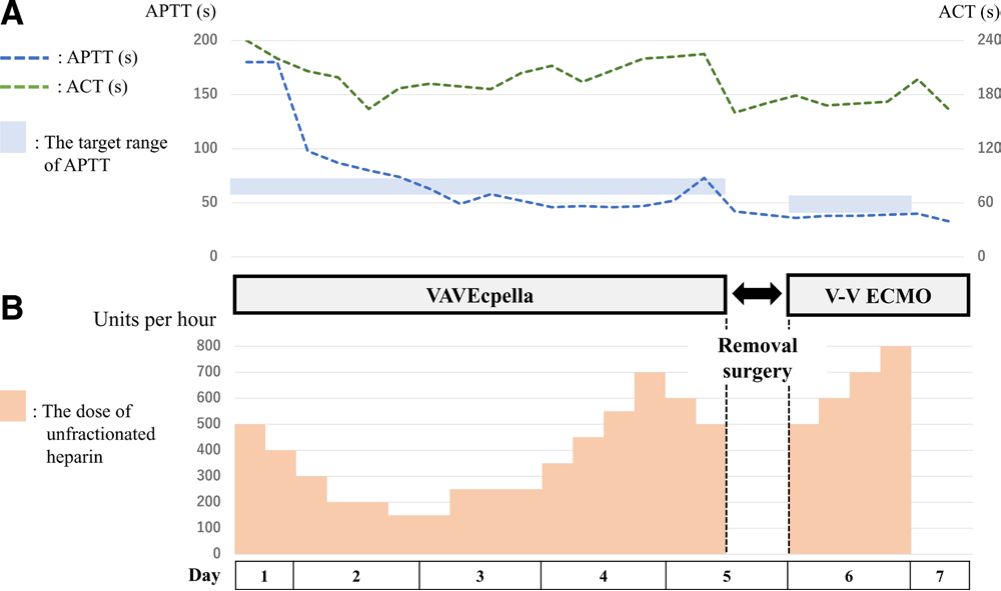
Anticoagulation management during mechanical circulatory support. (A) Figure shows the values of APTT and ACT. Both parameters were measured 3 to 4 times/d. During VAVEcpella support, the target APTT range was 60 to 70 s; during V-V ECMO support, it was 40 to 60 s. (B) The administered dosage of unfractionated heparin (units/h) is shown. The dosage was adjusted according to the APTT values. Heparin infusion was temporarily discontinued during surgery to change the MCS configuration. ACT = activated clotting time, APTT = activated partial thromboplastin time, MCS = mechanical circulatory support, s = second, VAVEcpella = veno-arteriovenous extracorporeal membrane oxygenation and Impella, V-V ECMO = veno-venous extracorporeal membrane oxygenation.

**Figure 5.**
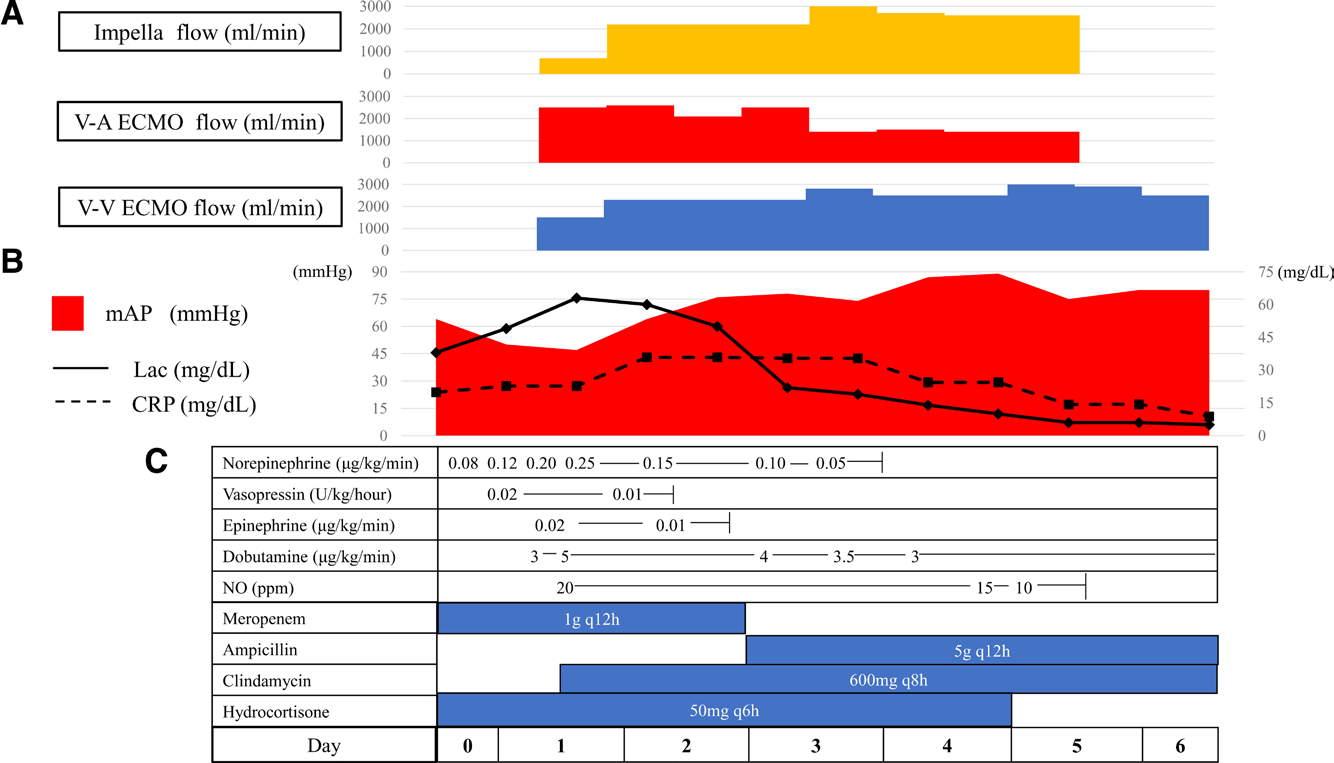
The clinical course from admission (day 0) to day 6. (A) Course of mechanical circulatory support devices consisting of Impella (Impella CP^®^) and V-AV ECMO. V-A ECMO flow means arterial line flow of V-AV ECMO. V-V ECMO flow is the return flow of the venous line of V-AV ECMO. MCS was introduced on the day after admission (day 1), and Impella CP^®^ and V-AV ECMO arterial line were removed on day 5. (B) Course of mean blood pressure (mAP), lactate (Lac), and C-reactive protein (CRP). After the introduction of MCS devices, mAP was increased and lactate level peaked out. (C) Course of the dose of vasoactive drugs, inhaled nitric oxide (NO), antibiotic, and hydrocortisone. CRP = C-reactive protein, Lac = lactate, mAP = mean blood pressure, MCS = mechanical circulatory support, NO = nitric oxide, V-AV ECMO = veno arteriovenous extracorporeal membrane oxygenation, V-A ECMO = veno-arterial extracorporeal membrane oxygenation, V-V ECMO = veno-venous extracorporeal membrane oxygenation.

Seventeen hours after admission, follow-up echocardiography revealed significant deterioration of LV wall motion (ejection fraction: 15%), raising the suspicion of SICM. Continuous intravenous dobutamine was initiated, but circulatory failure persisted, leading to cardiac arrest. Cardiopulmonary resuscitation achieved return of spontaneous circulation after 2 minutes and V-A ECMO was initiated. Due to severe hypotension and impaired oxygenation of the native lungs caused by pneumonia, we increased the V-A ECMO flow. However, echocardiography confirmed that the aortic valve remained closed, raising concerns about the formation of LV thrombus and further worsening of pulmonary congestion. To reduce afterload, an Impella CP^®^ was introduced. Additionally, to prevent differential hypoxia caused by the increased Impella flow, we added an additional venous return cannula to the ECMO circuit, resulting in a VAVEcpella configuration (Figs. 1D and 2B, C). Coronary angiography revealed no significant stenotic lesions and myocardial biopsy ruled out inflammatory infiltrates or secondary cardiomyopathy (Fig. 3C), indicating that the cardiac dysfunction was attributable to SICM. Anticoagulation with unfractionated heparin was aimed at an activated partial thromboplastin time (APTT) of 60 to 70 seconds (Fig. 4). After adding venous return to the ECMO circuit, right ventricular (RV) dilatation and worsening right heart failure were observed. Therefore, we increased the dose of dobutamine and initiated inhaled nitric oxide therapy. These interventions improved perfusion from the right ventricle to the left ventricle. The flow of the Impella increased, resulting in an increase in blood pressure (Fig. 5). Lactate levels steadily improved on the day after initiation of mechanical support (day 2) and C-reactive protein begun to decline on day 3, leading to the discontinuation of vasoactive and inotropic agents. On day 5, the echocardiographic evaluation showed gradual improvement in LV wall motion (ejection fraction: 45%), though hypoxia (PaO_2_/FiO_2_ ratio: 80) persisted. We then simultaneously removed the arterial line of the ECMO and the Impella support, changing the ECMO circuit from a VAVEcpella system to a veno-venous ECMO (V-V ECMO) system. Following the change in MCS configuration, the target APTT range was adjusted to 40 to 60 seconds (Fig. 4). On day 6, prone positioning therapy was administered in conjunction with high positive end-expiratory pressure (12–14 cm H₂O) to enhance oxygenation, which had been compromised due to bilateral atelectasis. On day 7, both pupils were unexpectedly dilated and a CT scan revealed extensive intracerebral hemorrhage. Neurosurgical evaluation confirmed irreversible brain damage, leading to transition to supportive care. Despite improved lung aeration (Fig. 2D) and successful weaning from V-V ECMO on day 9, progressive bradycardia from cerebral edema led to cardiac arrest and death on day 11.

Ethical approval was waived by our institutional ethics committee. Written informed consent was obtained from the patient’s family for the publication of this case and associated images.

## 3. Discussion

A case of STSS secondary to pneumonia due to group A streptococcal infection, which was complicated by severe respiratory failure and severe cardiac dysfunction due to SICM was treated with VAVEcpella, allowing prompt withdrawal of MCS.

MCS for refractory septic shock in adults remains controversial.^[7]^ Causes of circulatory failure in sepsis include distributive shock and cardiogenic shock due to SICM. In distributive shock, the primary pathophysiology involves decreased vascular resistance and increased capillary permeability, which limits the efficacy of mechanical support such as V-A ECMO. However, in cardiogenic shock due to SICM, V-A ECMO can effectively replace the reduced cardiac output and provide powerful circulatory support.^[9]^ Ling et al reported superior outcomes in septic shock patients with LVEF below 20% on V-A ECMO compared to those with LVEF above 35%, highlighting its utility in severe cardiac dysfunction.^[8]^ Additionally, cardiac function in SICM often recovers within a week, as in the present case where the LV ejection fraction improved from 15% to 45% within 5 days, in contrast to myocardial infarction, dilated cardiomyopathy, or myocarditis, thereby highlighting the role of ECMO as an effective bridging strategy.

In cases of severe LV dysfunction, retrograde arterial perfusion by V-A ECMO can restrict aortic valve opening and exacerbate pulmonary congestion. Under such circumstances, the addition of Impella device, which continuously drains blood from the left ventricle into the ascending aorta, for LV unloading is often necessary. In the acute phase, there may be limitations in increasing Impella flow; however, it can still be used effectively as a LV vent. When using Impella, antegrade flow may increase the risk of differential hypoxia, particularly in patients with compromised native lung function and respiratory insufficiency. In such cases, adding venous return to V-A ECMO can be an effective strategy to mitigate this risk. Although minimizing the configuration of MCS is generally preferable, in cases of STSS, rapid hemodynamic deterioration often necessitates the use of the most advanced and aggressive support, such as a combination of VAVEcpella. This approach can protect end organ function and provide valuable time for antibiotic therapy and local infection control measures. In addition, a comprehensive hemodynamic simulation study using MCS showed that VAVEcpella helps mitigate cardiac injury in the setting of biventricular dysfunction by reducing myocardial workload while maintaining total systemic flow and global oxygenation.^[14]^

Assessment of RV function is essential when utilizing a VAV-ECpella configuration in patients with SICM. Since SICM has been reported to involve biventricular failure,^[4]^ impaired RV function may result in inadequate LV filling, potentially leading to suction events with Impella. Furthermore, the addition of venous return to VA-ECMO may exacerbate RV failure. To support RV function, therapeutic strategies may include optimization of inotropic support, inhaled nitric oxide therapy, and increasing ECMO drainage flow and adjusting the amount of venous return, as in the present case.

Gandhi et al reported complications including bleeding (57.2%), stroke, hemolysis, infection, and limb ischemia in patients treated with V-A ECMO in combination with Impella.^[15]^ While data on VAVEcpella are lacking, complication rates may be comparable or higher. Sepsis-related coagulation activation exacerbates coagulopathy and complicates anticoagulation management.^[16]^ Therefore, the management of anticoagulation therapy for the MCS in septic shock is expected to be more complex than in cardiogenic shock. In this case, the patient received anticoagulation therapy via a continuous intravenous infusion of unfractionated heparin. This infusion was guided by a target APTT range and referring to activated clotting time values. However, after switching the MCS configuration from VAVEcpella to V-V ECMO and implementing prone positioning therapy, the patient experienced an intracranial hemorrhage. This may have been related to the use of high positive end-expiratory pressure during prone positioning, which might increase intracranial pressure due to potential inhibition of cerebral venous drainage.^[17]^ It may also be related to an increased heparin dosage compared to earlier in the course. As shown in Figure 4, APTT and activated clotting time values diverged over time, and the correlation between heparin dose and APTT changed as well. These results imply that the coagulation-fibrinolytic system may undergo dynamic alterations in response to systemic inflammation and changes in MCS configuration. When conventional anticoagulation monitoring is insufficient, viscoelastic assays, such as thromboelastography (TEG), may help more accurately assess coagulation status.^[18]^ There is no standardized approach to anticoagulation management in septic patients on advanced ECMO systems, which warrants further investigation.

The VAVEcpella configuration complicates the circuit. This requires the medical team to consistently share information about the source and volume of blood flowing through each access point. Additionally, measures to prevent pressure ulcers and enable the early detection of bleeding or neurological complications are indispensable. However, these requirements are associated with substantial costs in terms of healthcare expenditure and manpower, both of which remain challenges in clinical practice.

## 4. Conclusion

VAVEcpella provides potent respiratory and circulatory support and may serve as an effective salvage therapy for sepsis-induced cardiopulmonary failure, including severe respiratory failure due to pneumonia and circulatory failure due to SICM, particularly in cases such as STSS.

## Acknowledgments

We would like to thank Dr Shinya Shimizu, Department of Cardiology, Nagoya University, for his assistance in preparing the illustration in Figure 1.

## Author contributions

**Conceptualization:** Wataru Otsuru, Masaaki Nishihara.

**Supervision:** Masaaki Nishihara, Tomohiko Akahoshi.

**Visualization:** Wataru Otsuru, Masaaki Nishihara.

**Writing** – **original draft:** Wataru Otsuru, Masaaki Nishihara.

**Writing** – **review & editing:** Wataru Otsuru, Masaaki Nishihara, Kiwamu Hatakeyama, Takeshi Iyonaga, Takeo Fujino, Takafumi Sakamoto, Yuji Shono, Kohtaro Abe, Akira Shiose, Tomohiko Akahoshi.
